# Aflatoxin B1 levels, dietary exposure and cancer risk assessment in sesame and nut-based foods in Türkiye

**DOI:** 10.1007/s12550-025-00594-1

**Published:** 2025-06-09

**Authors:** Meryem Aydemir Atasever, Mukaddes Beyza Güler İnce, Betül Alkan Polat, Hayrunnisa Özlü, Mustafa Atasever

**Affiliations:** 1 Department of Food Hygiene and Technology, Faculty of Veterinary Medicine, Atatürk University, Erzurum, Türkiye; 2 Department of Medical Services and Techniques, Vocational School of Health Services, Erzincan Binali Yıldırım University, Erzincan, Türkiye; 3 Graduate School of Natural and Applied Sciences, Atatürk University, Erzurum, Türkiye

**Keywords:** Mycotoxins, Food contamination, Estimated daily intake, Margin of exposure, Public health

## Abstract

Aflatoxin B1 (AFB1), a highly toxic mycotoxin commonly found in oilseeds and nuts, poses serious public health and economic risks. Türkiye's climatic conditions are conducive to AFB1 contamination, particularly in sesame- and nut-based foods. This study investigated the presence of AFB1 in 100 widely consumed samples of tahini, tahini halva, hazelnut paste, and peanut paste. AFB1 was detected in 67% of the samples, with exceedances of the European Commission (EC) limit (2 µg/kg) notably high in tahini halva (80%) and peanut paste (60%); in contrast none of the hazelnut paste samples surpassed the legal threshold. The findings indicated that no samples of hazelnut paste exceeded the legal limits set by the EC. However, 4 tahini (13.33%), 24 tahini halva (80%), and 12 peanut paste (60%) samples exceeded the EC limits. Dietary exposure estimates were calculated based on lower bound (LB), middle bound (MB), and upper bound (UB) contamination scenarios. Margin of exposure (MOE) values for tahini halva were consistently below the safety threshold of 10,000, suggesting a possible health concern. The estimated annual incidence of hepatocellular carcinoma (HCC) due to AFB1 exposure ranged from 0.000888 to 0.00219 cases per 100,000 adults, remaining below European Food Safety Authority’s (EFSA) reference risk levels. These findings underscore the need for targeted surveillance and stricter regulatory controls on sesame-based products, particularly tahini halva, within national food safety frameworks.

## Introduction

Food safety and security are critical issues for both national and international organizations. Currently, the most pressing concerns focus on microbial and chemical hazards in food. Among the various food components, mycotoxins have gained significant global attention due to contamination by fungi (Sebaei et al. 2020). Additionally, global climate change is expected to lead to more severe droughts, reduced precipitation, and temperature increases of 2–3 °C in the Mediterranean region, including Türkiye (Daou et al. 2022; Aytekin Sahin et al. 2024). These changes may heighten the risk of mycotoxin contamination in this area (González-Curbelo and Kabak 2023).

Mycotoxins, primarily produced by fungi such as Aspergillus, Penicillium, Fusarium, and Alternaria, have a wide range of toxic effects. These effects include cytotoxicity, immune system modulation, estrogenic effects, genotoxicity, and carcinogenicity, which pose significant health risks (WHO 2023). Aflatoxins (AFs), a specific type of mycotoxin, are secondary metabolites produced mainly by certain toxigenic strains of Aspergillus, particularly A. flavus and A. parasiticus (Martinez-Miranda et al. 2019). Among all known types of AFs, aflatoxin B1 (AFB1), B2 (AFB2), G1 (AFG1), and G2 (AFG2) are the most commonly occurring natural toxins found in food and feed crops (EFSA 2020). AFB1 is particularly noteworthy as it is the most prevalent and potent genotoxin and carcinogen (Group 1) found in nature, as classified by the International Agency for Research on Cancer (IARC 2012).

Among all potential chemical hazards, AFB1 is the most potent. AFs are considered one of the most significant food safety concerns worldwide. AFB1, in particular, is associated with a high risk of developing hepatocellular carcinoma (HCC) in both humans and animals (WHO 2016).

The Rapid Alert System for Food and Feed (RASFF) recorded a total of 4,458 notifications from 2023 to 2024. Among these notifications, 209 fell under the categories of “Nuts, Nut Products and Seeds” and “Mycotoxins”. Out of these, only 10 (4.8%) were attributed to total AF, while 13 (6.2%) were linked to Ochratoxin A. The majority, 186 notifications (89.0%), were associated with AFB1 (RASFF 2024).

Determining the effects of toxic substances, such as mycotoxins, on an organism is known as risk assessment. A crucial component of this process is exposure assessment, which involves evaluating mycotoxin levels in food and consumption patterns. Conducting an exposure assessment allows for the identification of mycotoxins that may pose health risks to individuals and the public. The estimated values obtained from dietary exposure assessments can be used to develop strategies to reduce these risks (Huong et al. 2016; Aytekin Sahin et al. 2024).

Recently, the European Food Safety Authority (EFSA) and the Joint FAO/WHO Expert Committee on Food Additives (JECFA) have recommended using the margin of exposure (MOE) approach to assess carcinogenic and genotoxic substances (EFSA 2005). The MOE is a tool used in risk assessment to address potential health concerns from chemical contaminants in food and feed when it is unsuitable to establish a health-based guidance value (Mihalache et al. 2024). The MOE value can guide the implementation of measures to reduce exposure to harmful substances. An MOE level below 10,000 indicates a potential public health risk (EFSA 2005).

Tahini (or tahini sauce, tahini paste), also known as sesame seed paste, is made from hulled sesame seeds that are toasted and ground. It is a well-known condiment in the Middle East (Abu-Jdayil et al. 2002). Tahini halva (or helva) is a traditional product that adds sugar and other necessary ingredients to tahini paste and is generally consumed for breakfast in Türkiye. Likewise, peanut paste and hazelnut paste are consumed in Türkiye, especially for breakfast. As it is known, all these products are among the risky product groups in terms of AFB1 contamination.

Dietary risk assessments for AFs are crucial for guiding the implementation of measures to reduce associated risks and ensure food safety. Although there are few studies on hazelnuts and peanut paste, no studies have been conducted to evaluate mycotoxin contamination in tahini and tahini halva in the Turkish market. This study was conducted to determine AFB1 contamination in nut paste and sesame-based products produced in Türkiye, to estimate dietary exposure and risk assessment.

## Material and methods

### Sampling

In 2023, a total of 100 samples, including tahini, tahini halva, hazelnuts, and peanut paste, were collected from Erzurum Province, Türkiye. Although the samples were collected from Erzurum Province, the products selected represent nationally distributed brands that are widely available across Türkiye. All samples were obtained from large-scale national supermarket chains that operate throughout the country. Therefore, the selected products reflect the most commonly consumed sesame and nut-based food brands in Türkiye, increasing the representativeness of the findings despite the regional sampling. They were transported to the laboratory in a cooler with dry ice and stored at 4 °C until analysis.

### Determination of AFB1

An enzyme-linked immunosorbent serologic assay (ELISA) was employed to analyze the concentrations of AFB1 in samples of tahini, tahini halva, hazelnuts, and peanut paste using the RIDASCREEN® Aflatoxin B1 test kit (Aflatoxin B1 30/15, Art. No: R1211; R-Biopharm AG, Darmstadt, Germany). This approach utilizes a single-use RIDA® Aflatoxin immunoaffinity column (R-Biopharm AG) for effective sample clean-up before analyzing AFB1. The column is especially effective for cleaning challenging samples, such as nuts, herbs, spices, and tea leaves. This column featured a gel suspension with covalently bound monoclonal antibodies. The antibodies were specifically designed to target AFB1, B2, G1, and G2. As the AFs present in the sample flowed through the column, they attached to the monoclonal antibodies, while all other components were eliminated (Macri et al. 2021). This method ensures improved accuracy and reliability in results.

The extraction procedure began with 5 g of homogenized sample, which was extracted using 25 mL of methanol:water (70:30, v/v) by shaking for 30 min at room temperature. The extract was then centrifuged at 3000 rpm for 10 min, and the supernatant was filtered. An aliquot of the extract was diluted 1:10 with distilled water to ensure compatibility with antibody-based binding before being applied to the immunoaffinity column. The column was then washed and eluted according to the ELISA kit manufacturer’s instructions. Then the sample (1 mL) was passed slowly and continuously through the immunoaffinity column at a flow rate of approximately 1 drop/s to prevent gel compression and potential loss of aflatoxin. After the permeated solution was discarded, the column was rinsed with 10 mL of distilled water, and the liquid was discarded again. Some air was introduced into the column to ensure complete removal of the remaining liquid. To achieve complete elution of AFs, 0.5 mL of pure methanol was carefully passed through the column. This step was repeated if the eluent passed too quickly. All traces of eluent were collected by thoroughly pushing air through the column. The purification of the extract using immunoaffinity columns enhanced both specificity and sensitivity, improving accuracy by ensuring the interaction between the antigen and antibody.

After the cleanup process, AFB1 was detected. To test for it, 50 μL of the eluent, which contains the toxin, was mixed with 450 μL of distilled water. The testing followed the instructions provided by the manufacturer. The test kit included AFB1 standards at levels of 0, 5, 10, 20, 40, and 80 μg/kg. Samples with AFB1 levels lower than the assay's minimum detection limit were marked as negative for AFB1.

$$\text{Limit of detection }(\text{LOD}) = 1 \mu \text{g}/\text{kg}$$

### Estimated daily intake

When estimating dietary exposure, data on mycotoxin occurrence in foods and individual food consumption patterns are evaluated together. In these assessments, the most commonly applied approaches are estimation models that consider lower bound (LB) and upper bound (UB) values. However, according to the European Food Safety Authority (EFSA), to obtain more accurate and reliable results, left-skewed datasets should be processed using the substitution method, taking into account the proportion of left-censored values in the overall dataset (EFSA 2010). This method is recommended when the proportion of uncensored data is up to 60%. In this context, for samples containing a significant amount of left-censored data, the typical procedure involves assigning (1) zero for the LB estimate, (2) LOD/2 or LOQ/2 for the middle bound (MB) estimate, and (3) LOD or LOQ for the UB estimate. In this study, dietary exposure calculations for AFB1 were also carried out using the substitution method recommended by EFSA, since the proportion of uncensored data was below 60%. Total EDI values of AFB1 (ng kg^−1^bw day^−1^) were calculated using Eq. (1) (EFSA 2010):

1 $$\text{Total EDI }=\text{Di }\times \text{ Mi }/\text{W}$$

In this context, Di denotes the daily consumption (g/person/day) of sesame and nut-based foods sourced from Türkiye (FAOSTAT 2023). Mi reflects the average concentration of AFB1, measured in ng/g, while W represents body weight in kilograms (kg). For calculating the dietary exposure of adults to AFB1, a body weight (bw) of 73.7 kg was utilized, as recommended by the Turkish Statistical Institute (TUIK 2023).

### Risk characterization

To evaluate the risk, two distinct methodologies previously established by international regulatory agencies were utilized. These include the MOE approach proposed by the EFSA in 2005 (EFSA 2005) and a quantitative liver cancer risk assessment method put forth by the Food and Agriculture Organization and the World Health Organization in 2018 (WHO 2018). A more detailed description of both approaches can be found in the following sections.

### Margin of exposure approach

The MOE approach, introduced by the EFSA, is a method for assessing the risk of compounds that are both genotoxic and carcinogenic (EFSA 2005). This approach relies on a reference point typically derived from animal studies, which reflects a dose that leads to a low yet measurable increase in tumor formation-often quantified as a 1–10% rise above background levels in experimental animals. In the assessment of the MOE for AFB1, the 95% LB on the benchmark dose corresponding to a 10% extra risk (BMDL_10_) was utilized. This BMDL_10_ value of 0.4 µg/kg body weight per day has been identified as the most appropriate result by the EFSA (2020). According to established risk assessment criteria, an MOE value equal to or greater than 10,000 is interpreted as indicating a low risk to public health (EFSA 2005).

The MOE value is calculated using Eq. (2) given below:

2 $$\text{MOE} = \frac{\text{BMDL}_{10}}{\text{EDI}}$$

### Quantitative liver cancer risk approach

Additionally, the Joint FAO/WHO Expert Committee on Food Additives considered the incidence risk of HCC in its assessment of AFB1. The carcinogenic potency of AFB1 is estimated at 0.3 cancers per year for every 100,000 individuals exposed to 1 ng kg − ^1^ bwday^−1^ in hepatitis B surface antigen-positive (HBsAg +) individuals, and at 0.01 cancers per year for the same exposure level in hepatitis B surface antigen-negative (HBsAg −) individuals. For the Turkish population, the HBsAg + value of 4% was used, which is the rate reported in a recent study conducted by Özkan (2018) in Türkiye. The risk of AFB1-related liver cancer was calculated by the product of EDI and Pcancer (Eq. 3),

3 $$\begin{aligned}P_\text{cancer} &= 0.01 \times \% HBsAg^- + 0.3 \times \% HBsAg^+ \\P_\text{cancer} &= 0.01 \times 0.96 + 0.3 \times 0.04 = 0.022 \end{aligned}$$

Based on this carcinogenic potency, the annual risk of HCC incidence was calculated as follows (Eq. 4):

4 $$\text{HCC} = \text{EDI} \times P_{\text{cancer}}$$

### Statistical analyses

The data were analyzed using a one-way analysis of variance (ANOVA) conducted with SPSS version 19.0 (IBM, Chicago, IL, USA). The results are presented as mean ± standard deviation (SD), along with percentage distribution and frequency counts.

## Results and discussion

### AFB1 findings

**Fig. 1:**
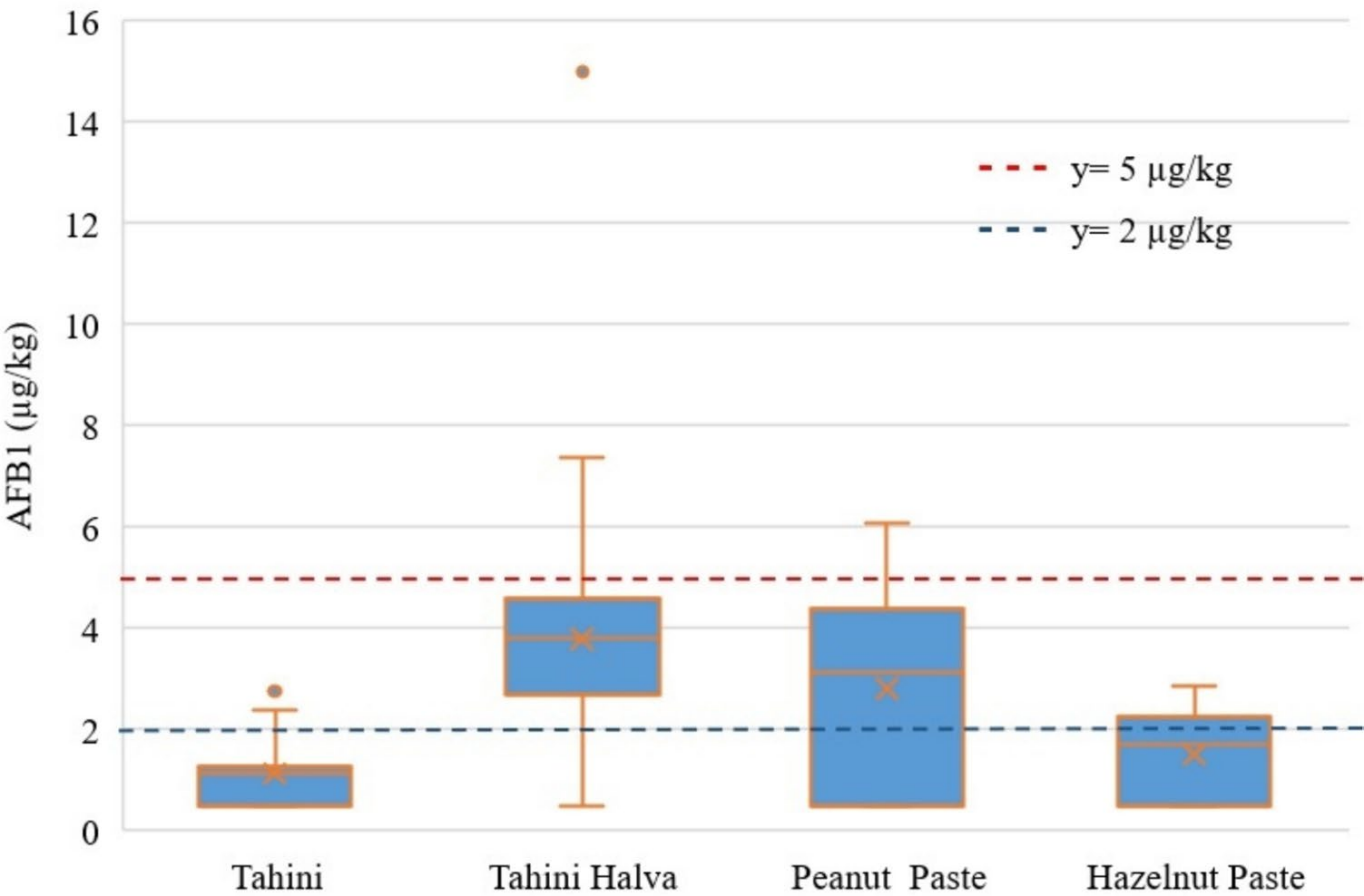
AFB1 distribution in tahini, tahini halva, hazelnut paste, and peanut samples

AFB1 values of tahini, tahini halva, hazelnuts, and peanut paste samples were presented in Table 1 and Fig. 1.

**Table 1:** AFB1 contamination in tahini, tahini halva, hazelnut paste, and peanut paste

		Tahini	Tahini Halva	Hazelnut paste	Peanut paste	Total
All samples						
Number		30	30	20	20	100
Frequency (%)	< LOD	40	20	40	35	33
	1–2 μg kg^−1^	46.67	ND	20	5	19
	> 2–5 μg kg^−1^	13.33	66.67	40	40	40
	> 5 μg kg^−1^	ND	13.33	ND	20	8
	LB	0.92	3,69	1.30	2.65	2.17
Mean (μg kg^−1^)	MB	1.12	3.79	1.50	2.82	2.34
	UB	1.32	3.89	1.70	3.00	2.50
Median (μg kg^−1^)		1.13	3.81	1.72	3.14	1.72
Positive Samples						
Number		18	24	12	13	67
Frequency (%)		60	80	60	65	67
Mean (μg kg^−1^)		1.54	4.61	2.17	4.07	3.24
Standard deviation		0.65	2.47	0.39	1.36	2.10
Median (μg kg^−1^)		1.60	4.14	2.20	4.08	2.81
Minimum (μg kg^−1^)		1.1	2.27	1.63	1.44	1.1
Maximum (μg kg^−1^)		2.81	15	2.86	6.09	15

*LB* lower bound, *MB* middle bound, *UB* upper bound, *LOD* Limit of detection, *ND* Not detected

The overall proportion of AFB1-contaminated (positive) samples of 67% was the sum of the following contributions: 18% in tahini, 24% in tahini halva, 12% in hazelnut paste, and 13% in peanut paste. The rate of AFB1-positive samples in individual product groups varied from 60% in tahini to 80% in tahini halva (Table 1). The high levels of AFB1 contamination in tahini halva may result from using tahini with high AFB1 contamination in its production. During peak production seasons, shriveled, immature, and relatively low-quality nut kernels are often selected for hazelnut and peanut paste production. The high incidence of AFB1 in samples of hazelnut and peanut paste likely stems from the choice of these lower-quality kernels.

Among the analyzed samples, two values were identified as statistical outliers (extreme value): the AFB1 level of 2.81 µg/kg in tahini and 15 µg/kg in tahini halva (Fig. 1). The samples, which exhibited a high deviation, exceeded the upper whisker limit in the Boxplot analysis and may indicate a potential contamination. In line with EFSA recommendations (EFSA 2021), these values were not excluded from the data analysis to maintain the reliability of the study, and this circumstance was explicitly highlighted.

According to the Turkish Food Codex (TFC) regulations, the maximum permissible level (MPL) for AFB1 is established at 5 µg/kg for groundnuts (peanuts) and hazelnuts, whether used as a single ingredient or in processed products intended for the final consumer or for use as a food ingredient. Moreover, the MPL for other oilseeds used as only ingredients or processed products from other oilseeds, placed on the market for the final consumer or use as an ingredient in food, lowering it to > 2 μg kg^−1^(TFC 2023). The MPL of AFB1 for tahini and tahini halva, the limit is > 2 μg kg^−1^, and for hazelnut paste, the limit is > 5 µg/kg, align with the limits set by both the European Commission (EC) and the TFC regulations. However, there is a difference regarding peanut paste; the EC regulation stipulates an aflatoxin limit of > 2 μg kg^−1^, whereas the TFC allows a higher limit of > 5 µg/kg. Figure 1 presents the sample rates of AFB1 that exceed the established legal limits in tahini, tahini halva, hazelnut paste, and peanut paste products (EC 2023; TFC 2023). While there were no samples in hazelnut paste samples exceeding the legal limits set by the EC and Türkiye, 4 tahini (13.33%), 24 tahini halva (80%) and 12 peanut paste (60%) samples exceeded the EC limits.

A total of 102 helva samples from Adana, Türkiye, were analyzed for AFB using thin-layer chromatography. Among them, AFB1 was not detected in plain and cacao helva but found in eight pistachio helva samples, with four exceeding Türkiye's legal limit of 5 µg kg⁻^1^ (Var et al. 2007).

Sebaei et al. (2020), who reported similar data to our study, determined that the concentrations of AFB1 in the contaminated black and white sesame samples were 0.84 to 2.08, and 1.02 to 2.17 μg/kg, respectively. 12% of the samples were below the MPL, and 21% exceeded the MPL of 2 μg/kg for AFB1 and 4 μg/kg for a total of AFB1, AFB2, AFG1, and AFG2 according to Egyptian standard specifications.

The Accra people in Ghana examined 53 different food brands, including pasta, rice, and cereal-based products, which were found to have the highest levels of AFB1, AFB2, and AFG1 at 65.77, 19.27, and 1.02 μg/kg, respectively (Kortei et al. 2019). Li et al. (2009) reported that 37% of sesame samples contained significant amounts of aflatoxin, with levels reaching up to 20 μg/kg.

A study on AFs in Chinese peanut butter and sesame paste found that AFB1 was the most commonly detected toxin, with levels reaching 68.51 μg/kg in 41 of 50 peanut butter samples and 20.45 μg/kg in 37 of 100 sesame paste samples. Among AFB1-positive samples, 37% of peanut butter and 19% of sesame paste exceeded Chinese regulations, while 2% of peanut butter and 32% of sesame paste surpassed the European Union (EU) limits (Li et al. 2009).

AFB1 contamination levels in halva (56), pistachio (71), almond (63), halva puri (39) samples, respectively 32 (%57), 45 (%63), 43 (%68), 21 (%54) were found. Among the levels above the EU limit (2 mg/kg), 11 (20%) halva, 23 (32%) pistachio, 34 (54%) almond, and 9 (23%) halva puri samples were included (Iqbal et al. 2013).

In a study conducted in Nigeria, melon (*n = *60) and sesame (*n = *60) seeds were most commonly contaminated with AFB1 and about 28 and 5% of melon and sesame, respectively, exceeded the 4 μg kg^−1^ total aflatoxin limit for oilseeds intended for direct human consumption in the EU (Esan et al. 2020).

### Health risk assessment

The average concentrations (LB, MB, UB) of AFB1 in sesame and nut-based foods in Türkiye and chronic exposure estimates calculated from per capita consumption patterns of these products are summarized in Table 2. Since daily consumption data for tahini in Türkiye were not available, risk analyses could not be calculated for this product.

**Table 2:** EDI, MOE and HCC findings due to AFB1 contamination in tahini halva, hazelnut paste, and peanut paste samples for the Turkish adult population

Samples		Tahini Halva	Hazelnut paste	Peanut paste
Daily intake* (g day^−1^)		1.99	1.75	0.8
Mean AFB_1_ (μg kg^−1^)	LB	3.69	1.30	2.65
	MB	3.79	1.50	2.82
	UB	3.89	1.70	3.00
EDI (ng kg^−1^ bw day^−1^)	LB	0.10	0.03	0.03
	MB	0.10	0.04	0.03
	UB	0.10	0.04	0.03
MOE	LB	4018	12,963	13,932
	MB	3911	11,234	13,067
	UB	3811	9912	12,304
Annual HCC incidence (cases per 100,000 persons per year	LB	0.00219	0.000679	0.000632
	MB	0.00225	0.000783	0.000673
	UB	0.002309	0.000888	0.000715

*FAOSTAT 2023, Point of departureof BMDL_10_: 0.4 µg kg^−1^ bw day^−1^, Average body weight (adult):73,7 kg

*LB* lower bound, *MB* middle bound, *UB* upper bound, *EDI* estimated daily intake, *MOE* margin of exposure, *HCC* hepatocellular carcinoma

In this study, the mean AFB1 contamination levels in tahini halva, hazelnut paste, and peanut paste ranged between 1.30 μg/kg and 3.89 μg/kg based on the LB, MB, and UB scenarios. The EDI values for the adult Turkish population were calculated to be between 0.03 ng/kg bw/day and 0.10 ng/kg bw/day across the same scenarios. Correspondingly, MOE values ranged from 3811 to 13,932, while the estimated annual incidence of HCC varied between 0.00219 and 0.000888 cases per 100,000 individuals (Table 2). The average MOE values calculated for the adult population in Türkiye were found to be below 10,000, particularly in tahini halva samples, suggesting a potential public health concern. In contrast, the associated risk appears to be lower in hazelnut paste and peanut paste samples (EFSA 2020).

The EFSA's 2020 report estimated that average dietary exposure to AFB1 for adults ranges from 0.22 to 0.49 ng kg^−1^bw day^−1^ (LB) and 1.35 to 3.25 ng kg^−1^bw day^−1^ (UB). For the 95 th percentile, the exposure ranges from 0.62 to 1.36 ng kg^−1^bw day^−1^ (LB) and 2.76 to 6.78 ng kg^−1^bw day^−1^ (UB) (EFSA 2020). The Scientific Committee on Food stated that exposure to AFs as low as 1 ng kg^−1^bw day^−1^ may increase the risk of liver cancer (EFSA 2007). Considering this information, it can be concluded that the EDI values determined in this study are low. Based on the mean potency estimates and a prevalence rate of 0.2%, the CONTAM Panel concluded that the cancer risk associated with average dietary exposure to AFB1 in adults ranges from 0.004 to 0.057 aflatoxin-induced cancers per 100,000 people per year. Additionally, utilizing upper-bound potential estimates and a prevalence rate of 7.6%, the same panel assessed the worst-case potential risk of cancer from dietary exposure to AFB1 in adults, estimating it to be between 0.019 and 0.286 aflatoxin-induced cancers per 100,000 people per year (EFSA 2020). In this study, the estimated annual risk of HCC associated with the consumption of tahini halva, hazelnut paste, and peanut paste among the adult Turkish population was found to be below the reference risk values established by EFSA.

Although the ELISA method employed in this study is widely recognized for routine mycotoxin screening due to its cost-effectiveness, ease of use, and broad accessibility, it has a relatively high limit of detection (1 µg/kg). This may lead to underestimation of AFB1 contamination, particularly in samples with low toxin levels, and thus, the actual dietary exposure might be higher than estimated. Moreover, ELISA is susceptible to analytical limitations such as matrix effects and potential cross-reactivity, which can introduce some degree of uncertainty in exposure assessment. Nevertheless, EFSA (2020) considers ELISA a suitable and valid method for dietary exposure and risk assessment. Supporting this, Serraino et al. (2019) and Udovicki et al. (2021) reported that the uncertainty linked to ELISA-based data is generally limited and unlikely to compromise the overall reliability of exposure estimates.

A review of the literature on AFB1 contamination in sesame and nut-based products reveals substantial variation across studies. While several investigations report findings comparable to those observed in our study, others indicate notably higher levels of contamination. This discrepancy reflects the inherent challenges of accurately assessing mycotoxin levels in different food matrices and emphasizes the importance of ongoing research to facilitate more robust and context-specific risk assessments.

In a study conducted in Türkiye, it was reported that 1 out of 202 hazelnut paste samples exceeded EU limits for AFB1 (0.5%), the average concentration (LB-UB) was 2.197–2.198 mg kg^_1^, and the contamination range was between 0.25–5.42 mg kg^_1^. For the adult Turkish population (15 + years age group), the average LB and UB exposure levels for AFB1 were 0.0106–0.0107 ng kg^−1^ bw day^−1^. MOE estimates for mean and 95 th percentile exposures to AFB1 for hazelnut paste were higher than 10,000, which indicates no potential health concern for Turkish adults. HCC for the Turkish population was 0.00023 cases per 100,000 people per year. The study indicates that the Turkish population is not under this toxicological risk when consuming hazelnut paste-containing food products (Şen and Civil 2022).

Santos et al. (2018) reported that 24.0% of 104 peanut samples collected in Brazil were found to be contaminated, with an average concentration of 13.4 μg/kg. Furthermore, twenty samples, representing 19.2%, exceeded Brazil's maximum permitted level for total aflatoxin. The estimated probable daily intake was 1.28 μg kg^−1^bw day^−1^, which surpassed the Provisional Maximum Tolerable Daily Intake of 0.001 μg kg^−1^bw day^−1^.

A study involving 792 food samples revealed that 27.1% exhibited detectable levels of AFs ranging from 0.07 to 262.63 μg kg⁻^1^. Among the various AFs detected, AFB1 emerged as the most prevalent across the different sample categories. Additionally, peanuts and rice were identified as the primary contributors to dietary exposure to AFs among residents of Zhejiang (Fang et al. 2022).

To evaluate aflatoxin exposure in Taiwan from peanuts and related products, 1,089 samples were collected between 2011 and 2017. The average contamination level of AFB1 was 2.40 μg/kg. Levels of peanuts from China, Indonesia, Thailand, the U.S., and the Philippines exceeded the safe limit of 10,000, suggesting no significant public health risk for most people (Lien et al. 2019).

Nugraha et al. (2018) revealed that AFB1 exposure from Indonesian corn and peanut consumption was a concern. MOE values for corn and peanut consumption in Indonesia generally fell below 10,000, and in some cases even below 1,000. In addition, the estimated number of liver cancer cases associated with AFB1 exposure was generally 0.1 cancer cases/per 100,000 people/age over 75 years.

In a study aimed at evaluating AFB1 concentration and health risk in peanut samples in China, 255 peanut samples were collected in 2020. Four point seven percent of the samples were found to be contaminated with AFB1. The MOE values of AFB1 ranged from 3,549 to 3,281 (LB to UB), which were lower than the safety threshold. At both the mean and the high (97.5 th) percentile intake levels, the liver cancer risk associated with AFB1 exposure was higher than the annual incidence of liver cancer in China (17.7 cases per 100,000 persons per year), indicating that peanut consumption could pose a significant cancer risk (Dong et al. 2023).

The contamination of AFs in 120 samples of sesame seeds, tahini, and tahini halva collected from Iran’s market was evaluated. The AFB1 concentration in sesame seeds, tahini, and tahini halva was in the ranges of 0.21–12.35, 0.23–5.81, and 0.27–3.56 μg/kg, respectively. The concentration levels of AFB1 in 10 (25%), 7 (17.5%), and 6 (15%) samples of sesame seeds, tahini, and tahini halva, respectively, were higher than the European regulations (2 μg/kg). As the percentiles 50 and 95 of MOE with AFB1 for sesame seed, tahini, and tahini halva were more than 10,000, it could be concluded that the intake of aflatoxin through the consumption of the mentioned products did not pose a remarkable cancer risk for adults (Heshmati et al. 2021).To mitigate the identified risks, targeted risk management strategies should be implemented, including improved pre-harvest practices, enhanced storage conditions to prevent fungal contamination, and more frequent routine sampling of high-risk products such as tahini halva. Given that tahini halva is a traditional food widely consumed by children in Türkiye, this population group may face increased dietary exposure, underscoring the need for protective regulatory measures and public awareness efforts.

## Conclusion

This study provides critical evidence of AFB1 contamination in sesame and nut-based products commonly consumed in Türkiye. Among the 100 nationally distributed samples analyzed, tahini halva and peanut paste exhibited notably high levels of AFB1, with a substantial proportion of samples exceeding the EC’s legal limits. In contrast, hazelnut paste samples remained within regulatory thresholds. Dietary exposure assessment revealed that while EDI levels were relatively low, the MOE values for tahini halva fell below the health-based guidance value of 10,000, indicating a potential public health concern. Despite the low estimated annual incidence of HCC, the findings underscore the need for enhanced risk management strategies targeting sesame-based products, particularly tahini halva. Regular monitoring, improved processing practices, and stricter regulatory oversight are essential to mitigate aflatoxin-related health risks. Future studies should incorporate larger, more diverse sample sets and employ advanced analytical techniques, alongside nationally representative consumption data, to refine risk estimates and inform public health policies.

## Limitations

Although the collection of samples exclusively from Erzurum Province may be considered a limitation, it is important to note that all products analyzed in this study were non-local items that are commercially available on a national scale throughout Türkiye. Therefore, the sampling strategy employed may mitigate this limitation by ensuring broader representativeness of the findings. The use of ELISA, while widely accepted for routine screening and compatible with risk assessment frameworks, is associated with a relatively high limit of detection (1 µg/kg), potentially leading to underestimation of low-level contamination and dietary exposure. Additional uncertainty may also result from matrix effects and possible cross-reactivity. While the ELISA method followed the manufacturer’s protocol and aligns with EFSA guidelines, detailed method validation, such as evaluation of measurement uncertainty, recovery, and precision, was not conducted, which may affect the reliability and reproducibility of the results. Moreover, the study focused solely on three aflatoxin-prone food products—tahini halva, hazelnut paste, and peanut paste—without including other major dietary sources such as cereals, dried fruits, and spices. As a result, the estimated dietary exposure and risk values presented here may not fully represent the total aflatoxin burden for the Turkish population. Future studies are encouraged to address these limitations by incorporating broader and more geographically representative sampling, applying more sensitive analytical techniques such as LC–MS/MS, and integrating comprehensive consumption data and uncertainty modeling to support more accurate and representative risk assessments.

## Data Availability

No datasets were generated or analysed during the current study.
